# Community carbon and water exchange responses to warming and precipitation enhancement in sandy grassland along a restoration gradient

**DOI:** 10.1002/ece3.5490

**Published:** 2019-09-17

**Authors:** Yayong Luo, Xiaoan Zuo, Yulin Li, Tonghui Zhang, Rui Zhang, Juanli Chen, Peng Lv, Xueyong Zhao

**Affiliations:** ^1^ Naiman Desertification Research Station Northwest Institute of Eco‐Environment and Resources Chinese Academy of Sciences Lanzhou China; ^2^ Laboratory of Stress Ecophysiology and Biotechnology Northwest Institute of Eco‐Environment and Resources Chinese Academy of Sciences Lanzhou China; ^3^ Urad Desert Steppe Research Station Northwest Institute of Eco‐Environment and Resources Chinese Academy of Sciences Lanzhou China

**Keywords:** net ecosystem productivity, precipitation, sandy grassland, warming

## Abstract

Temperature increasing and precipitation alteration are predicted to occur in arid and semiarid lands; however, the response mechanism of carbon and water exchange at community level is still unclear in semiarid sandy land. We investigated the responses of carbon and water exchanges to warming and precipitation enhancement along a sand dune restoration gradient: mobile sand dunes (MD), semifixed sand dunes (SFD), and fixed sand dunes (FD). The average net ecosystem productivity (NEP) and evapotranspiration (ET) between May and August increased by 98% and 59%, respectively, from MD to SFD, while they had no significant differences between FD and the other two habitats. Warming inhibited ecosystem NEP, ET, and water use efficiency (WUE) by 69%, 49% (*p* < .001), and 80%, respectively, in SFD, while it nearly had no significant effects in MD and FD. However, precipitation addition by 30% nearly had no significant effects on community NEP, ET, and WUE, except for warming treatment in FD. In general, precipitation addition of 30% may still not be enough to prevent drought stress for growth of plants, due to with low water holding capacity and high evaporation rates in sandy land. Temperature increase magnified drought stress as it increased evapotranspiration rates especially in summer. In addition, community NEP, ET, and WUE were usually influenced by interactions between habitats and temperature, as well as the interactions among habitats, temperature, and precipitation. Species differences in each habitat along the restoration gradient may alter climate sensitivity of sandy land. These results will support in understanding and the prediction of the impacts of warming and precipitation change in semiarid sandy grassland.

## Graphical abstract

Precipitation addition of 30% under these conditions may still not be enough to prevent drought stress for the growth of plants, due to sandy soil texture with low water holding capacity and high evaporation rates in semiarid sand land. Temperature increase magnified drought stress as it increased evapotranspiration rates especially in summer. These results will support in understanding and the prediction of the impacts of warming and precipitation change in semiarid sandy grassland.

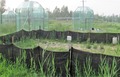

## 1 INTRODUCTION

Sandy land ecosystems play a significant role in carbon sequestration (Zuo et al., 2015) due to their large area in many parts of the world, especially in China. Results showed that sandy land has large carbon (C) sequestration potential under beneficial conditions (Miao et al., 2015), but may also turn into a large C source under unfavorable conditions such as overgrazing (Brunet & Larson‐Rabin, 2012). In addition, these areas are vulnerable to climate and land use change, leading to uncertainty in their contributions to regional and global carbon budgets (Brunet & Larson‐Rabin, 2012).

Climate change is expected to result in warmer temperature and changing precipitation pattern for most parts of the world (IPCC, 2013). Net ecosystem productivity (NEP) represents the balance between gross ecosystem productivity (GEP) and ecosystem respiration (ER), addtionally, the responses of GEP and ER to warming and increased precipitation are largely dependent on soil moisture and nutrient status in arid and semiarid regions (Huang, Li, & Padilla, 2015; Sponseller, 2007). Therefore, understanding how ecosystem carbon (C) and water exchange respond to warming and altered precipitation at the proper ecological scale is essential for our valuing of ecosystem processes.

Warming is expected to have a diverse and intense impact on biology ranging from cellular to the ecosystem level, such as plant photosynthesis and respiration, plant phenology, species distribution, and decomposition of soil organic matter (Lin, Xia, & Wan, 2010; Martinez et al., 2014). A meta‐analysis showed that warming significantly increased total ecosystem net primary production, photosynthesis, and respiration (Wu, Dijkstra, Koch, Penuelas, & Hungate, 2011). However, Shi et al. (2015) reported that warming decreased the turnover rate of the live C pool but increased the turnover rate of litter and fast soil C pool; as a result, warming decreased gross primary production and total ecosystem C. The effect of warming on optimum temperature, warming time, and water availability varies among ecosystems. At the temperature below the optimum level, warming contributes to plants; however, it was shown that temperature which is higher than optimal one for plant growth or photosynthesis produced adverse effects on plant photosynthesis, productivity, and water use efficiency (Bauweraerts et al., 2013; Song, Wang, & Lv, 2016). On the other hand, warming usually accelerates ecosystem respiration in the short term, but in the long term, responses of respiration to warming are less clear because most warming experiments are too short (Li et al., 2017; Rustad et al., 2001). Additionally, warming often increases evapotranspiration especially temperature at high level, which leads to a more severe water deficit and exaggerated the aridification or desertification of arid and semiarid areas (Maestre, Salguero‐Gomez, & Quero, 2012).

Altered precipitation regimes represent a sensitive and dramatic impact on plant photosynthesis, growth, and productivity (Salazar‐Parra et al., 2015), especially in arid and semiarid regions (Yue, Zhang, Zhao, Liu, & Ma, 2016). Increased precipitation is expected to be favorable to plant photosynthesis, growth, and species richness (Salazar‐Parra et al., 2015; Yue et al., 2016). Gross ecosystem productivity was found to be more sensitive to altered water availability than ecosystem respiration (ER; Niu et al., 2009); therefore, water addition may enhance NEP in temperate semiarid steppe. However, the overall effects of altered precipitation on NEP remain highly controversial, which may have been resulted from differences in climate type, soil texture, species composition, and root distribution (Huang et al., 2015; Koerner & Collins, 2014).

Precipitation alteration in the seasonal distribution, rainfall frequency, and intensity has profound impacts on plant growth and ecosystem carbon exchange by affecting soil infiltration and evaporation, etc. (Wilcox, von Fischer, Muscha, Petersen, & Knapp, 2015). Soil texture affects its water holding capacity and available water, which may affect strongly the availability of water and the resulting response of plant growth to precipitation. Plant growth is increased with rising precipitation in loam or clay soils with high water holding capacity. However, few studies have been conducted in semiarid or arid regions with sandy soil considering this issue.

Horqin sandy land is one of the most severely desertified regions of China. However, due to relatively higher annual precipitation (about 340 mm), the degraded vegetation of mobile dunes could gradually be restored after excluding grazing (Zhang, Zhao, Zhang, Zhao, & Drake, 2005; Zuo et al., 2015). Previous studies have documented that plant species richness, biomass, soil C, and N increased with vegetation succession from mobile dunes (MD) to semifixed dunes (SFD) and toward fixed dunes (FD; Li et al., 2012; Zuo et al., 2015). Yet, there is limited information about how community carbon and water exchange respond to warming and precipitation enhancement along the habitat gradient of sandy dune restoration.

In order to understand the impacts of projected changes in both warming and precipitation enhancement on the community carbon and water exchange of sandy grassland ecosystems, we conducted a warming and water addition experiment in sand dunes along a restoration gradient in northeastern China. We measured ecosystem C and water fluxes for 4 consecutive months that differed greatly in the amount of precipitation in the growing season. We hypothesized that (a) NEP would increase along a sand dunes restoration gradient; (b) warming would inhibit NEP; and (c) water addition would enhance NEP in temperate semiarid sand dunes.

## 2 MATERIALS AND METHODS

### 2.1 Site description

This study was conducted in a sandy grassland ecosystem of Horqin Sandy Land (42°55′N, 120°42′E; elevation approx. 360 m) in the northeast of Inner Mongolia, Northern China. The area has a strong temperate, semiarid continental monsoonal climate with a warm summer and a very cold winter. The mean annual precipitation is 343 mm, with more than 75% falling within the growing season from June to September. The mean annual temperature is approximately 7.0°C, with monthly mean temperatures ranging from a minimum of −13.0°C in January to a maximum of 23.7°C in July (Figure 2). The annual mean latent evaporation is 1,935 mm. The annual mean wind velocity is in the range of 3.2–4.1 m/s. The topography is characterized by sand dunes and interdunes. The sandy soil is vulnerable to wind erosion, and the sandy grassland is ecologically fragile and subject to desertification.

Three habitats were selected, which represent typical successional habitats (six replicate sites per habitat) along a restoration gradient of sand dune, including MD with <10% vegetation cover, SFD with 10%–60% vegetation cover, and FD with more than 60% vegetation cover (Zuo et al., 2015). These sites were located at 0.5–8 km distance from each other. The dominant pioneer species on MD is *Agriophyllum squarrosum*. In SFD, the dominant species is shrub *Artemisia halodendron* and forb *Corispermum macrocarpum*. Fixed dunes are dominated by *Artemisia scoparia*. *Setaria viridis* and *Eragrostis pilosa* distributed in three habitats along a restoration gradient, but the quantity is diverse.

### 2.2 Air temperature and precipitation manipulations

**Figure 1:**
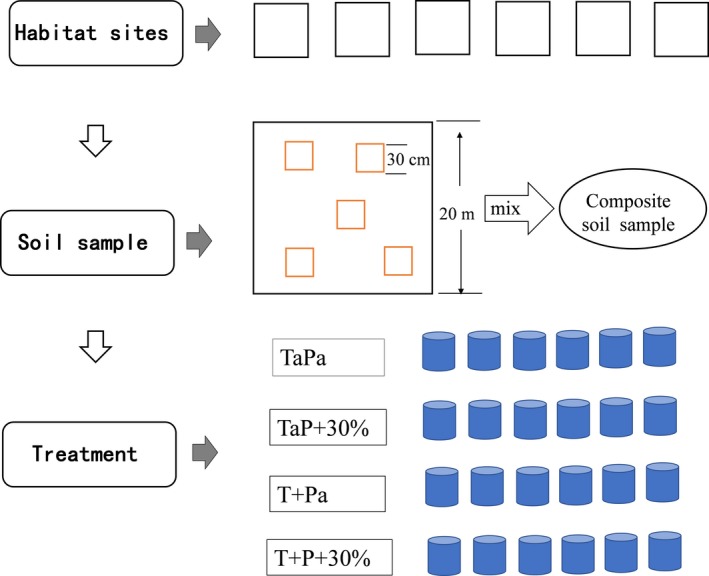
Basic outline of experiments for each sand dune habitat at different restoration stages (MD, SFD, and FD). For each habitat, six replicated 20 × 20 m sites with flat topography (slope <5°) were selected to soil sample. Surface 5 cm soil was collected in five 30 × 30 cm quadrats for representative seed banks in each site. The soil samples from each habitat at six sites were mixed thoroughly to produce a composite sample. Twenty‐four PVC tubes (30 cm diameter and 30 cm depth) were prepared for pot experiment for each habitat. Each tube filled the top 5 cm with the composite seed bank samples, after filling 25 cm of sand in advance below. The experiment included two temperature treatments: control (Ta) and warmed (T+). Each combined with two precipitation treatments: ambient precipitation (Pa) and precipitation increased by 30% (P + 30%), for treatments included TaPa, TaP + 30%, T + Pa, and T + P + 30%. Each treatment had 6 tubes of repetition for each sand dune habitat

The experiment included two temperature treatments: control (Ta) and warmed (T+). Each combined with two precipitation treatments: ambient precipitation (Pa) and precipitation increased by 30% (P + 30%; Figure 1), which is about difference between average precipitation in the years of abundant water and average rainfall in the past 55 years in the study area. The P + 30% treatment involved adding water to the plots after each precipitation.

Open‐top hexagonal chambers (OTC) were used to increase air temperature (T+) in experimental plots. The chamber design was modified from that of the International Tundra Experiment (Marion et al., 1997), with each of the six component walls of a chamber made of aluminum support frames with attached clear glass. The length of the six outside edges of the aluminum frame wall was 2 m bottom edge and 2 m height. The walls of the assembled chamber were oriented at a 60° angle and had a height of 1 m with 1 m top edge. Air temperature and relative humidity were measured in and out of OTC synchronously every half hour between July 16 and August 15.

### 2.3 Experimental design

In each 20 × 20 m site with flat topography (slope <5°), surface 5 cm soil was collected in five 30 × 30 cm quadrats for representative seed banks. The soil samples from each habitat at different restoration stages (MD, SFD, and FD) were mixed thoroughly to produce a composite sample, respectively. Seventy‐two PVC tubes (30 cm diameter and 30 cm depth) were prepared for pot experiment. Each tube was on a tray and contained 25 cm depth sandy soil without seeds in advance on 14 April 2015, then filled the top 5 cm with the composite seed bank samples (Figure 1). Excess water was allowed to drain through holes in the bottoms of the trays. To ensure the germination of plant seeds, 1 kg water (about 15 cm precipitation) was added into each pot at the beginning. Each treatment had six tubes of repetition for each sand dune habitat, precipitation, and temperature treatment, and 72 pots were cultivated in total. To avoid seed entry in ambient pots, pots was surrounded by ambient by shade net of 70 cm height in a circle.

### 2.4 Measurements

Air temperature and precipitation data were collected from a weather station about 150 m from the experiment field. NEP and evapotranspiration (ET) were measured using LI‐840A CO_2_/H_2_O Gas Analyzer connected with an assimilation chamber (30 cm diameter and 35 cm height). Two fans were installed into assimilation chamber to ensure consistent air distribution. The ratio of NEP to ET was calculated to determine water use efficiency (WUE).

### 2.5 Statistical analysis

Repeated measures ANOVA was used to investigate (a) differences in carbon and water exchange parameters under different habitats (H), temperature (T), and precipitation (P) conditions; (b) changes in carbon and water exchange parameters over 4 months from May to August; and (c) examine the effects of month, habitats, warming, precipitation enhancement, and their interactions on these parameters. Due to month had significant interaction with other factors, then conduct ANOVA analysis at each month. The data of NEP and ET were log‐transformed to meet the requirements of data normal distribution in ANOVA. All statistical procedures were carried out using SPSS 19.0 software. The general linear model (GLM) process was used to examine their effects and interactions on carbon and water exchange parameters at each month. For habitats at different restoration stages, the effect of habitat on these parameters was tested with one‐way ANOVA at each month. Student's *t* test analyses on these parameters were performed for testing significant differences (*p* < .05) between warming and control, and between ambient precipitation and precipitation increased by 30% in each habitat, and on each month measurements, respectively.

## 3 RESULTS

### 3.1 Air temperature and precipitation

**Figure 2:**
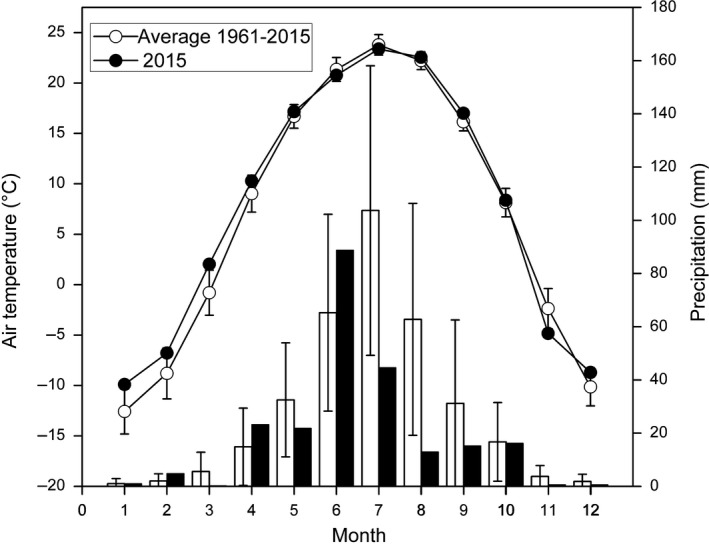
Comparison of the monthly average air temperature and monthly total precipitation. Blank circle and column represent mean ± *SD* every month between 1961 and 2015; solid circle and column represent average values in 2015

**Figure 3:**
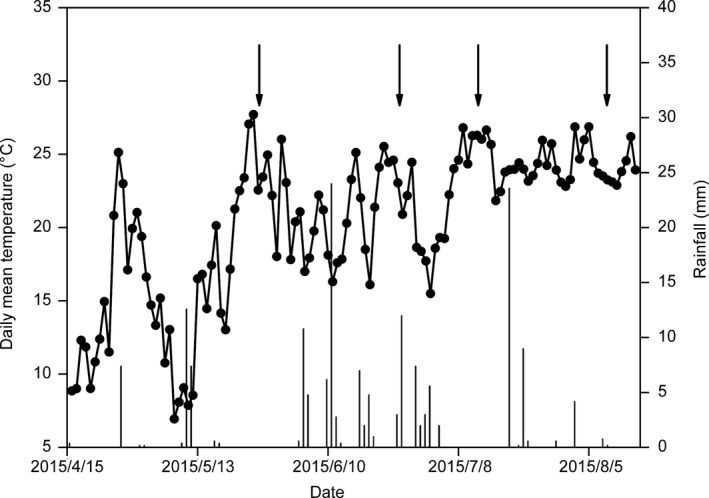
Daily rainfall and temperature through experiment, blank circle and column represent daily mean temperature and rainfall, respectively. Arrow indicates the date of measuring carbon and water exchange (May 26, June 27, July 13, August 10)

A comparison of the monthly average air temperature and monthly total precipitation between 2015 and 55 years (1961–2015) mean in Horqin Sandy land is shown in Figure 2. Air temperature during 2015 growing season was within one standard deviation of the 55 years mean for all months, which was warmer than normal in May and September, but cooler between June and August. In 2015, the total precipitation was 230 mm, lower than 343 mm annual mean precipitation in the 55 years. The relatively low precipitation in July and August in 2015 resulted in total growing season precipitation 38% lower in 2015 (183 mm) than that of the long‐term average (297 mm). There were several relatively dry periods except June during the growing season in 2015. The rainfall and air temperature during the experimental periods are shown in Figure 3. Compared to control, air temperature increased 3.3°C and the relative humidity decreased 6.9% in OTC between July 16 and August 15.

### 3.2 Community carbon and water exchange character along a restoration gradient

**Figure 4:**
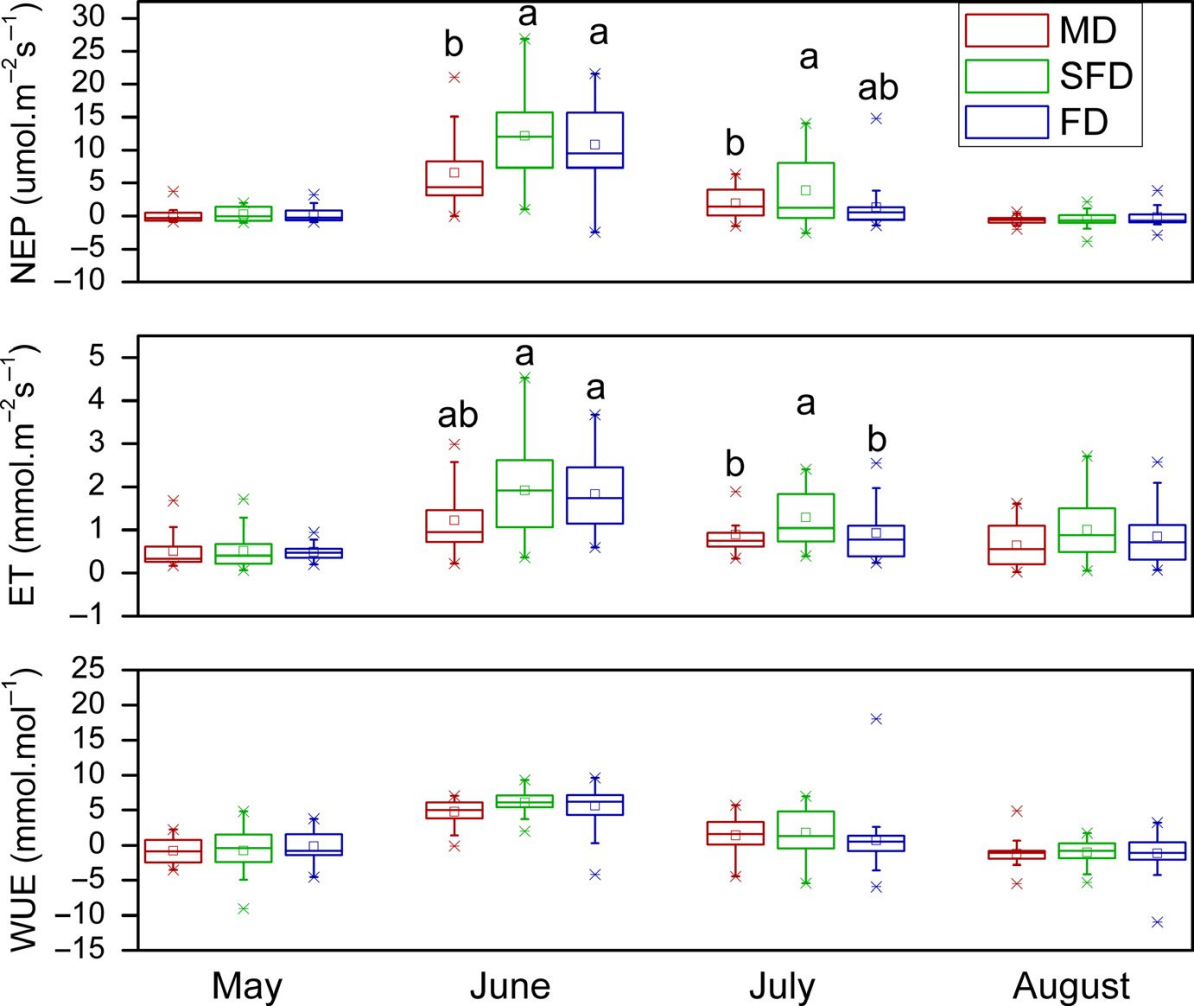
Community net ecosystem productivity (NEP), evapotranspiration (ET), and water use efficiency (WUE) along a restoration gradient (MD: mobile sand dune, SFD: semifixed sand dune, FD: fixed sand dune). Red, green, and blue show mobile dunes (MD), semifixed dunes (SFD), and fixed dunes (FD), respectively. □ and represent mean and outlier (1% and 99%). Different letters in mean values indicate the statistical difference of same variable among different habitats at *p* < .05

Through the growing season, NEP (*p* = .01) and ET (*p* = .04), which effect size represented by partial estimated‐squared with 95% confidence intervals $\eta_{p}^{2}$ equaled .158 and .118, respectively, differed significantly along the restoration gradient of sand dunes (i.e., from MD to FD); nevertheless, they were statistically significant only between MD and SFD (Table 1, Figure 4). The average community NEP and ET between May and August increased by 98% (*p* = .006) and 59% (*p* = .022), respectively, from MD to SFD. WUE had no significant differences between May and August among three habitats (*p* = .495, $\eta_{p}^{2}$ = .028; Table 1, Figure 4).

**Table 1:** *F* value and *p* value of repeated measures ANOVA from May to August with habitats (H), temperature (T), and precipitation (P)

Source	*df*	NEP			ET			WUE		
		*F*	*p*	$\eta_{p}^{2}$	*F*	*p*	$\eta_{p}^{2}$	*F*	*p*	$\eta_{p}^{2}$
Habitats (H)	2	5.06	.010	.158	3.43	.040	.118	0.71	.495	.028
Temperature (T)	1	12.52	.001	.188	14.32	<.001	.219	16.74	<.001	.255
Precipitation (P)	1	1.01	.320	.018	1.56	.218	.030	2.73	.105	.053
Month (M)	3	72.23	<.001	.806	47.93	<.001	.746	137.13	<.001	.737
H × T	2	5.85	.005	.178	3.95	.025	.134	1.41	.254	.054
H × P	2	0.07	.934	.003	0.18	.836	.007	1.23	.302	.048
T × P	1	2.80	.100	.049	0.82	.369	.016	10.68	.002	.179
H × T × P	2	4.00	.024	.129	3.89	.027	.132	6.78	.003	.217
M × H	6	4.23	.001	.193	2.24	.045	.118	0.86	.517	.034
M × T	3	28.08	<.001	.618	19.47	<.001	.544	22.70	<.001	.317
M × P	3	0.98	.410	.053	1.30	.287	.073	0.83	.469	.017
M × H × T	6	1.71	.126	.088	1.13	.353	.063	1.12	.352	.044
M × H × P	6	0.87	.520	.047	1.95	.08	.105	3.12	.009	.113
M × T × P	3	3.38	.025	.163	6.38	.001	.281	3.59	.018	.068
M × H × T × P	6	1.71	.125	.088	0.96	.454	.055	0.71	.633	.028

Note

Effect size represented by partial estimated‐squared ($\eta_{p}^{2}$) with 95% confidence intervals.

Abbreviations: *df*, degrees of freedom; ET, evapotranspiration; NEP, net ecosystem productivity; WUE, water use efficiency.

There are significant differences among the months for NEP (*p* < .001) with effect size represented by partial estimated‐squared with 95% confidence intervals ($\eta_{p}^{2}$ = .806), ET (*p* < .001, $\eta_{p}^{2}$ = .746), and WUE (*p* < .001, $\eta_{p}^{2}$ = .737). Interaction between months and temperature had significant effects on NEP (*p* < .001, $\eta_{p}^{2}$ = .618), ET (*p* < .001, $\eta_{p}^{2}$ = .544), and WUE (*p* < .001, $\eta_{p}^{2}$ = .317), as well as interactions among months, temperature, and precipitation (*p* = .025, *p* = .001, *p* = .018) with effect size ($\eta_{p}^{2}$ = .163, $\eta_{p}^{2}$ = .281, $\eta_{p}^{2}$ = .068), respectively. Significant interaction between months and habitats was observed in NEP (*p* = .001, $\eta_{p}^{2}$ = .193) and ET (*p* = .045, $\eta_{p}^{2}$ = .118), but it had no significance on WUE (*p* = .517, $\eta_{p}^{2}$ = .034). Additionally, WUE (*p* = .001) was significantly affected by the interaction among months, habitats, and precipitation with effect size ($\eta_{p}^{2}$ = .118), but the effects on NEP (*p* = .520, $\eta_{p}^{2}$ = .047) and ET (*p* = .08, $\eta_{p}^{2}$ = .105) were not significant (Table 1).

Net ecosystem productivity and WUE in June and July were apparently higher than those in May and August when NEP was close to 0 across the three different desertification stages. NEP and WUE were positive between May and July, but negative in August in the three habitats. In June, NEP was lower significantly in MD than those found in FD (*p* = .027) and SFD (*p* = .004), respectively, while it had no significant differences between FD and SFD. Community NEP in the FD and SFD was 1.9 and 1.7 times of that in the MD, respectively. Community ET was lower significantly in MD than those found in SFD (*p* = .009), but it had no significant differences between FD and other two habitats in June. Community ET in the FD and SFD was 1.9 and 1.5 times of that in the MD, respectively. In July, NEP was higher significantly in SFD than those in FD (*p* = .036), but it had no significant differences between MD and other two habitats. Community NEP in the SFD and MD was 2.9 and 1.4 times of that in the FD, respectively. Community ET was higher significantly in SFD than those in MD (*p* = .030) and FD (*p* = .046), respectively, while it had no significant differences between MD and FD in July. Community ET in the FD and SFD was 1.4 and 1.04 times of that in the MD, respectively. Community NEP and ET had no significant difference in May and August in the three habitats along the restoration gradient, respectively (Figure 4).

### 3.3 Community carbon and water exchange responses to warming

**Figure 5:**
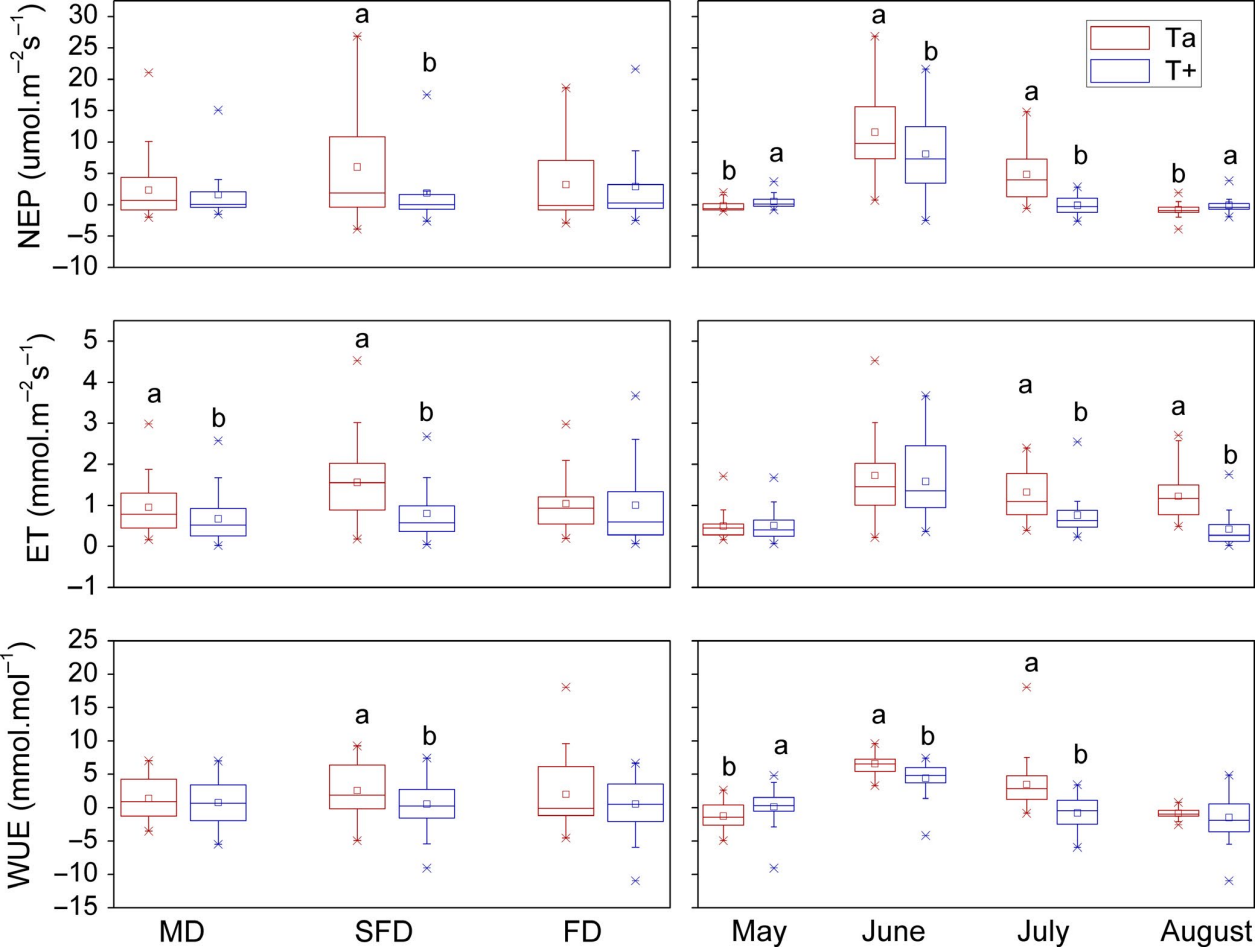
Effects of warming on NEP, ET, and WUE. Red and blue show ambient temperature (Ta) and increasing temperature (T+), respectively. □ and 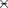 represent mean and outlier (1% and 99%). Different letters in mean values indicate the statistical difference of same variable between warming and ambient treatment at *p* < .05. The left panels show the overall (i.e., May–August) effects of the treatment on the different dune types. The right panels show the overall (i.e., MD, SFD and FD) effects of the treatment at each month

As a whole, warming significant inhibited community NEP (*p* = .001) with effect size represented by partial estimated‐squared with 95% confidence intervals ($\eta_{p}^{2}$ = .188), ET (*p* < .001, $\eta_{p}^{2}$ = .219), and WUE (*p* < .001, $\eta_{p}^{2}$ = .255). Average community NEP, ET, and WUE between May and August for three habitats decreased by 46% (*p* = .001), 25% (*p* < .001), and 82% (*p* < .001) after warming, respectively (Table 1, Figure 5). Warming inhibited NEP, ET, and WUE by 69% (*p* = .003), 49% (*p* < .001), and 80% (*p* = .013), respectively, in SFD, and decreased ET by 29% (*p* = .032) in MD, while it had no significant effects in FD (Table 1, Figure 5). Although community NEP and WUE in May and August when NEP was close to 0, warming facilitated significantly community NEP in May (*p* = .013) and August (*p* = .018), respectively, and facilitated community WUE in May (*p* = .022), but had no significant effect in August. However, warming decreased NEP and WUE by 30% (*p* = .033) and 34% (*p* < .001), respectively, in June, and decreased NEP (*p* < .001) and WUE (*p* < .001) to negative value in July. Community ET was decreased by 42% (*p* < .001) in July and 65% (*p* < .001) in August, respectively, after warming, but it had no significant effects in May and June (Figure 5).

### 3.4 Community carbon and water exchange responses to precipitation enhancement

**Figure 6:**
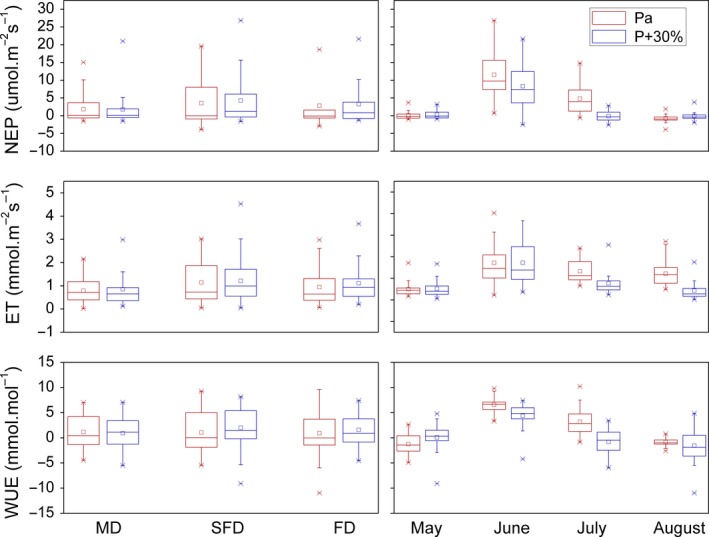
Effects of precipitation enhancement on NEP, ET, and WUE. Red and blue show ambient precipitation (Pa) and precipitation enhancement by 30% (P + 30%), respectively. □ and 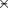 represent mean and outlier (1% and 99%). The left panels show the overall (i.e., May–August) effects of the treatment on the different dune types. The right panels show the overall (i.e., MD, SFD and FD) effects of the treatment at each month

Precipitation enhancement by 30% affected community carbon and water exchange differently, compared to warming. It had no significant effects on community NEP (*p* = .320) with effect size represented by partial estimated‐squared with 95% confidence intervals ($\eta_{p}^{2}$ = .018), ET (*p* = .218, $\eta_{p}^{2}$ = .030), and WUE (*p* = .105, $\eta_{p}^{2}$ = .053) between May and August for three habitats. They also had no significant differences to precipitation enhancement, for each habitat and month, respectively (Table 1, Figure 6), while average community NEP, ET, and WUE for three habitats increased by 23%, 21%, and 72% between May and August after increasing precipitation by 30%, respectively (Figure 6).

### 3.5 Community carbon and water exchange responses to warming and precipitation enhancement

**Figure 7:**
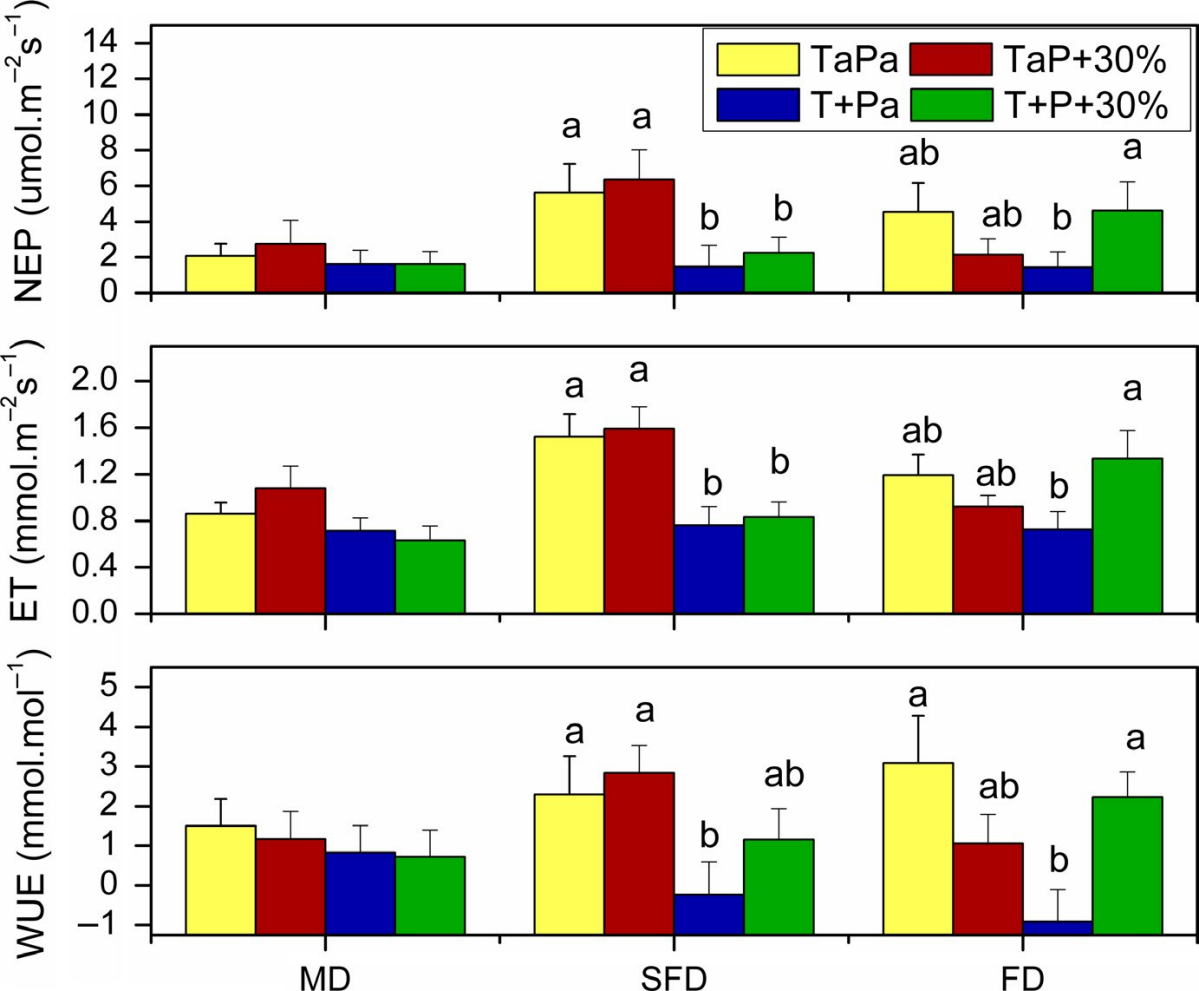
Effects of warming and precipitation enhancement on NEP, ET, and WUE. Yellow, red, blue, and green show control, ambient temperature and increased precipitation, increased temperature and ambient precipitation, and increased temperature and increased precipitation, respectively. Different letters in mean values indicate the statistical difference of same variable among the four treatment *p* < .05. Values are means + *SE*

Through the growth season, habitats, temperature, and their interactions significantly influenced NEP (*p* = .01, *p* = .001, *p* = .005) and ET (*p* = .04, *p* < .001, *p* = .025), respectively, although warming effects on these carbon and water exchange parameters were not significant (all *p* > .05) in MD (Figure 7). In addition, warming and its interaction with precipitation effected on WUE (*p* < .001, *p* = .002), with effect size represented by partial estimated‐squared with 95% confidence intervals ($\eta_{p}^{2}$ = .255, $\eta_{p}^{2}$ = .179), respectively. Interactions among habitats, temperature, and precipitation had significant effects on NEP (*p* = .024), ET (*p* = .027), and WUE (*p* = .003; Table 1).

Warming inhibited NEP, ET, and WUE by 74% (*p* = .038), 50% (*p* = .019), and 110% (*p* = .041), respectively, for ambient precipitation, and 65% (*p* = .046), 48% (*p* = .008), and 59% (*p* = .179) for precipitation enhancement by 30% in SFD, respectively. Warming also decreased NEP, ET, and WUE in MD for two precipitation treatments, as well as for ambient precipitation in FD, respectively, although the effects were not significant (all *p* > .05). However, the warming effects were positive for precipitation enhancement by 30% in FD (Figure 7). That is, the negative effects of warming were divergence in FD in different precipitation treatment.

Precipitation enhancement by 30% had positive effects on NEP, ET, and WUE for both temperature treatment in SFD, although the effects were not significant (all *p* > .05). The effects were positive for control temperature except for WUE and negative for warming treatment in MD, although the effects were not significant (all *p* > .05). On the contrary, the effects were negative for control temperature and significant positive in FD. That is, the effects of precipitation enhancement were different among sandy dunes along the restoration gradient in different temperature treatment, respectively (Figure 7).

## 4 DISCUSSION

Sandy land has large carbon sequestration potential under beneficial conditions. However as sandy land is sensitive to environmental factors, this potential is effected by changes in these factors. Restoration of mobile sand dunes (MD) to fixed sand dunes (FD) could occur under beneficial climate or via human protection (Miao et al., 2015). However, mechanisms of carbon and water exchange responses to climate change in sandy ecosystem would differ from other ecosystems due to the soil texture, which is characteristic for its high quick infiltration rate and high maximum temperatures. These factors strengthen water and heat stresses by warming, but weaken the beneficial affects of precipitation enhancement on growth of the herbaceous plants (Luo, Zhao, Zuo, Li, & Wang, 2017).

### 4.1 Habitat effects along a restoration gradient

In this study, net ecosystem productivity (NEP) and evapotranspiration (ET) had no significant differences in May and August among the three habitats along the restoration gradient, respectively (Figure 4). But they were the highest in semifixed sand dunes (SFD) and lowest in MD in June and July, respectively (Figure 4). The results showed smaller differences in community NEP among the three habitats, and even higher NEP in SFD than FD, were found in July. These results are inconsistent with hypothesis 1 which stated that NEP will increase along a sand dunes restoration gradient, which may be due to changes in species composition in pot experiment. On one hand, dominant species *Artemisia scoparia* in the wild habitat was replaced by *Chenopodium acuminatum*, *Setaria viridis* (SV), and *Eragrostis Pilosa* in the pot experiment in FD. Especially, *A. scoparia* dominated in wild FD habitat usually germinated in the previous year, and the seedlings were removed in the seed bank pot. On the other hand, symbiotic species, such as *Setaria viridis* (SV) and *Eragrostis Pilosa*, were prosperous in the pot experiment in MD. They are C_4_ species with higher growth and photosynthesis (Table 2). In other words, in the pot experiment, the original FD dominant species in the wild habitat was decreased remarkably, but C_4_ species were prosperous in MD.

**Table 2:** Dominant species of each treatment along the restoration gradient

Treatment	MD	SFD	FD
TaPa	SV 77.2, DC 10.9, EP 10.3	CM 77.4, SV 22.2	CA 43.2, SV 19.0, EP 17.6
TaP + 30%	SV 45.0, AS 20.1, CM 17.2	CM 90.2, SV 7.0	EP 47.9, ASC 21.1, SV 20.0
T + Pa	SV 67.5, CM 29.4	CM 99.3	SV 20.0, TM 16.8, CA 15.4, TT 13.4
T + P + 30%	SV 71.0, AS 14.4	CM 91.3	TT 38.7, CA 32.2, EP 20.7

Abbreviations: AS, *Agriophyllum squarrosum*; ASC, *Artemisia scoparia*; CA, *Chenopodium acuminatum*; CM, *Corispermum macrocarpum*; DC, *Digitaria ciliaris*; EP, *Eragrostis pilosa*; SV, *Setaria viridis*; TM, *Tragus mongolorum*; TT, *Tribulus terrestris*.

Compared with other characteristics of plant, soil, and litter along the due gradient in the previous studies, which have documented that plant species richness, biomass, litter mass, and C and N storage in plant biomass, litter and soil increased with vegetation succession from mobile dunes to semifixed dunes then in fixed dunes (Li et al., 2012; Zhang et al., 2005; Zuo et al., 2015). For example, total ecosystem C and N storage increased by 1.9, 4.8 and 3.3, 15.7 times, respectively, with the conversion from mobile dune to semifixed dune and fixed dune (Zuo et al., 2015).

### 4.2 Warming effects

Warming could promote the growth of plants because of increasing the growing season (Xu et al., 2015), increased availability of soil nutrients (Dawes, Schleppi, Hattenschwiler, Rixen, & Hagedorn, 2017), photosynthetic enhancement (Rustad et al., 2001; Wu et al., 2011), and changes of water use strategy (Li, Lin, Taube, Pan, & Dittert, 2011). However, warming may promote the mineralization of soil nutrients; as a result, plant will distribute more material to assimilate organs and reduce the distribution of material underground (Dawes et al., 2015), which may result in decreasing the root:shoot ratio of some plant species (Xiao, Zhou, & Ceulemans, 2003).

In the present study, warming facilitated significantly community NEP and WUE in May and August, but inhibited them significantly between June and July (Figure 5), which partly supports hypothesis 2 that warming would inhibit these factors. The result found in this study is similar to that found by Zelikova et al. (2015). They showed that differential daytime/night‐time warming (1.5/3°C) for 8 years increased vegetation cover and greenness early in the growing season, but often had a negative effect during the middle of the summer in a semiarid grassland. As so, it is suggested that warming may have different effects in different stages of the growing seasons. In addition, the increase of temperature can cause plants suffering more severe water stress as a result of increased evapotranspiration rates, which can ultimately lead to a decrease in photosynthesis and retarding of the growth of plants in water restricted areas (Bai et al., 2010; Luo et al., 2017). More severe water stress may explain the negative effect of community NEP between June and July in this study. The negative effects were greater than the positive effects in May and August, so warming inhibited community NEP in the growing season as a whole (Figure 5). Unexpectedly, warming increased the NEP, ET, and WUE for precipitation enhancement by 30% treatment in FD. This could be because that relative dominance of *Tribulus terrestris* and *Chenopodium acuminatum* with higher growth rate increases (Table 2). Plant growth may respond differently among species after warming which is consistent with previous results (Dawes et al., 2015). For example, woody biomass increased greatly, but the biomass of graminoids, forbs, and nonvascular plants decreased after 6 years of soil warming (at +4°C) of alpine vegetation near the tree line (Dawes et al., 2015).

### 4.3 Precipitation enhancement effects

Precipitation contributes to soil moisture directly by adding water to the system, which influences plant growth strongly (Yang et al., 2011), especially in semiarid regions (Song et al., 2016). Precipitation enhancement may increase photosynthesis (Song et al., 2016), plant community coverage (Wu et al., 2011), and extend the growing season (Wertin, Reed, & Belnap, 2015) to promote the growth of plants. However, precipitation enhancement may distribute more material to assimilate organs, and thus, roots were inhibited. In addition, there are some reports that plants are not sensitive to water boost (Luo et al., 2017; Yang et al., 2016).

In this study, it was shown that precipitation of plus 30% had no significant effects on community NEP and ET between May and August, respectively (Figure 6). One possible reason is that interaction among precipitation, habitat, and temperature had significant effects on NEP (*p* = .024) with effect size represented by partial estimated‐squared with 95% confidence intervals ($\eta_{p}^{2}$ = .129), ET (*p* = .027, $\eta_{p}^{2}$ = .132), and WUE (*p* = .003, $\eta_{p}^{2}$ = .217; Table 1). Precipitation enhancement by 30% had positive effects on NEP, ET, and WUE for both temperature treatment in SFD, for control temperature except WUE in MD, and for warming treatment in FD, while it had negative effects on NEP, ET, and WUE for warming treatment in MD, and for control temperature in FD, respectively, although these effects were mostly insignificant. Therefore, the divergent effects among different dunes and between two temperature treatment counteract the effects of precipitation increments (Figure 7). Other causes could be soil moisture and soil texture in this study, which was carried out only in a year with roughly half of the long‐term mean growing season precipitation amount, thereby creating significant water stress, and even 30% of additional water to the system may have not been enough to prevent a drought stress for plant growth. This may have been exacerbated by the effect of the soil texture on water availability, as soil texture strongly affects drainage and evaporation, which in turn affects water availability to plants. The soil water availability is more strongly and directly related to plant growth than to the amount of precipitation (Guo et al., 2016). In addition, soil has a low water holding capacity and the soil water content decreased rapidly after rainfall events in sandy land, due to large soil saturated hydraulic conductivity and high evaporation (Yao, Zhang, Zhao, & Liu, 2013). Therefore, soil water content in shallow layers (0–30 cm) was found to decrease in about 10% within 24 hr after precipitation events ranging from 5.7 to 110.1 mm in Horqin sandy land (Liu et al., 2015). Moreover, the slight and insignificant influence of precipitation addition on community NEP may have resulted from the limited space in this study or different plant species response.

Particularly, precipitation enhancement by 30% increased NEP (*p* = .043), ET (*p* = .039), and WUE (*p* = .024) significantly for warming treatment in FD (Figure 7). The reason may be the change of community structure (Table 2). Therefore, hypothesis 3 that water addition would enhance NEP also was partly supported. That is, the effects of precipitation enhancement were positive in SFD, only for control temperature in MD and for warming treatment in FD, although the effects were not significant mostly (*p* > .05; Figure 7). Therefore, the effects of precipitation enhancement were divergence among sandy dunes along the restoration gradient in different temperature treatment, respectively. These results support a theory, that is, successional change in species composition alters climate sensitivity of grassland productivity (Shi et al., 2018).

## 5 CONCLUSION

In conclusion, the average community NEP and ET between May and August increased by 98% (*p* = .006) and 59% (*p* = .022), respectively, from MD to SFD, while they had no significant differences between FD and the other two habitats. Unexpectedly, NEP was higher in SFD than FD (Table 1, Figure 4). Warming inhibited community NEP, ET, and WUE in the growing season for three habitats, although the effects had no significant differences mostly in MD and FD (Table 1, Figures 5 and 6). However, warming increased the NEP, ET, and WUE for precipitation enhancement by 30% treatment in FD, although the effects had no significant differences (Figure 7). Precipitation enhancement had no significant effects on community NEP, ET, and WUE mostly, except for warming treatment in FD. Particularly, precipitation enhancement by 30% increased NEP, ET, and WUE significantly for warming treatment in FD (Figure 7). Species differences in each habitat along the restoration gradient may alter climate sensitivity of sandy land.

## CONFLICT OF INTEREST

None declared.

## AUTHOR CONTRIBUTIONS

Yayong Luo designed the experiments, performed the data analyses, and wrote the manuscript. Xiaoan Zuo conceived the experiments. Yulin Li helped perform the analysis with constructive discussions. Tonghui Zhang put forward constructive suggestions on the revision of the manuscript. Rui Zhang revised the manuscript. Juanli Chen performed the experiments. Peng Lv performed the experiments. Xueyong Zhao guided the research and approved the final version.

## Data Availability

All data supporting this study are provided as [Link], [Link], [Link], [Link] accompanying this paper. Data obtained 3 documents. The document “Difference between precipitation and temperature in 1961–2015 and 2015” included data for Figure 2. The document “Precipitation and temperature during the experiment in 2015” included data for Figure 3. The document “responses to warming and precipitation enhancement along habitats” included data for Figures 4, 5, 6, 7. We not only have registered in a public archive‐Dryad, but also submitted our data according to your guide. Data package title: Data from: Community carbon and water exchange responses to warming and precipitation enhancement in sandy grassland along a restoration gradient. Journal: Ecology and Evolution. Provisional DOI: https://doi.org/10.5061/dryad.5h058nc. Data files: Difference between precipitation and temperature in 1961–2015 and 2015. Precipitation and temperature during the experiment in 2015. Responses to warming and precipitation enhancement along habitats.
