# Supplementary material for: Community carbon and water exchange responses to warming and precipitation enhancement in sandy grassland along a restoration gradient
Source: Ecol Evol. 2019 Sep 17;9(19):10938–49. doi: 10.1002/ece3.5490 (PMC6802028; doi:10.1002/ece3.5490)
Supplement: Supplementary file 1 [file ECE3-9-10938-s001.docx]

Comparison of the monthly average air temperature and monthly total precipitation. It includes the monthly data of mean ± SD between 1961 and 2015, as well as average values for each month in 2015. The data was shown in figure 1.

Daily rainfall and temperature through experiment.

Community net ecosystem productivity (NEP), evapotranspiration (ET) and water use efficiency (WUE) along a restoration gradient (MD: mobile sand dune, SFD: semi-fixed sand dune, FD: fixed sand dune). Red, green and blue show mobile dunes (MD), semi-fixed dunes (SFD) and fixed dunes (FD), respectively. The experiment included two temperature treatments: control (Ta) and warmed(T+). Each combined with two precipitation treatments: ambient precipitation (Pa), and precipitation increased by 30% (P+30%). The community net ecosystem productivity (NEP), evapotranspiration (ET) and water use efficiency (WUE) were measured for each month between May and August.

Thank you for your submission! Your data package has been forwarded to Dryad curation staff, and you have been sent a confirmation email containing a provisional DOI for your data package. Once your submission has been reviewed and approved, the DOI becomes permanent and can be used to cite your data package. You should hear from us within two business days. (Large or complex submissions may require a longer response time).

**Data from: Community carbon and water exchange responses to warming and precipitation enhancement in sandy grassland along a restoration gradient**

**Data Files**

Difference between precipitation and tempe... 2015

Precipitation and temperature during the e... 2015

responses to warming and precipitation enh...itats

The Fig 1 was added formally, so the other figures was postponed in turn. The figure legend and position, as well as table, were added in the maintext.

Your submission to the Dryad repository titled "Data from: Community carbon and water exchange responses to warming and precipitation enhancement in sandy grassland along a restoration gradient" is now being processed by the curatorial team. You should receive an update within two business days. (Large or complex submissions may require a longer response time).

YOUR DRYAD DOI

Your data package has been assigned a unique identifier, called a DOI. This DOI is provisional for now, but may be included in the article manuscript. It will be fully registered with the DOI system when your submission has been approved by Dryad curation staff.

Data package title: Data from: Community carbon and water exchange responses to warming and precipitation enhancement in sandy grassland along a restoration gradient

Journal: Ecology and Evolution

Provisional DOI: doi:10.5061/dryad.5h058nc

Data files: Difference between precipitation and temperature in 1961-2015 and 2015

Precipitation and temperature during the experiment in 2015

responses to warming and precipitation enhancement along habitats

PAYMENT

If you were asked to enter a credit card number, your card has been verified, but will be not be charged until the curatorial team accepts your submission. You will receive a confirmation and a receipt when your card is charged.

Please email us at help@datadryad.org if you have any questions or concerns.

The Dryad Team
